# High intensity focused ultrasound (HIFU) in the treatment of breast fibroadenomata: a feasibility study

**DOI:** 10.1186/2050-5736-3-S1-O77

**Published:** 2015-06-30

**Authors:** Mirjam Peek, Muneer Ahmed, Julie Scudder, Arnie Purushotham, Ashutosh Kothari, Hamed Hisham, Tibor Kovacs, Sarah McWilliams, Sarah Pinder, Bennie ten Haken, Michael Douek

**Affiliations:** 1 King’s College London, London, United Kingdom; 2 Guy’s and St. Thomas’, London, United Kingdom; 3 University of Twente, Enschede, Netherlands

## Background/introduction

Breast fibroadenomata (FAD) are the most common breast lesions in woman. For palpable lesions, three options are available: reassurance (with or without follow-up), vacuum assisted mammotome (VAM) or surgical excision. High intensity focused ultrasound (HIFU) is a non-invasive ablative technique in which an ultrasound (US) beam propagates through tissue as a high-frequency pressure wave and is focused onto targeted tissue which elevates the temperature of the focused area without causing damage to the adjacent tissues. HIFU is normally applied to the whole lesion. In this trial, we performed circumferential HIFU treatment to isolate the fibroadenoma from its blood supply. Primary end points of this trial included the decrease in treatment time and short-term complication rate.

## Methods

Patients (age ≥18 years) were recruited with symptomatic fibroadenomata which had to be visible on US (grade U2/U3). In patients older than 25 years, a fine needle aspiration cytology (FNAC) or core needle biopsy (CNB) was performed to confirm the diagnosis of a FAD. Patients were treated using the US guided - TH-one Echopulse device (Theraclion, France) under local anaesthesia (1:1, 1% Lignocaine with adrenaline and 0.25-0.50% Chirocaine).

## Results and conclusions

From December 2013, 13 patients with symptomatic fibroadenomata underwent circumferential HIFU treatment. Seven patients opted for HIFU treatment due to pain or discomfort. Average treatment time for approximately 61 sonications (SD, 17 sonications), was 36 minutes (SD, 12 minutes). Circumferential treatment reduced treatment time by an average of 44% (SD, 21%). A two-sample T-test assuming unequal variances showed a significant reduction in treatment time (P = 0.005, two-tailed).

Post-treatment follow-up at 2 weeks showed reduced pain in six out of seven patients with resolution of pain in three of these. An additional patient developed new pain after two weeks. Short-term complications were erythema of the skin (n=4), ecchymosis (n=4), temporarily numbness of the skin (n=1) and a first-degree skin burn (n=1).

Circumferential HIFU ablation of fibroadenomata is feasible with a significant reduction in treatment time.

